# Ferroptosis suppressor protein 1-mediated ferroptosis suppression in sepsis: non-canonical antioxidant pathways, inflammatory regulation, and therapeutic perspectives

**DOI:** 10.3389/fimmu.2026.1892904

**Published:** 2026-08-07

**Authors:** Yonghuan Tian, Zaiyong Li, Tian Tian, Hudie Zhu, ManHong Zhou, Xianping Long, Song Wang

**Affiliations:** 1 Department of Emergency, Affiliated Hospital of Zunyi Medical University, Guizhou, Zunyi, China; 2 Department of Emergency, KweiChow Moutai Hospital, Guizhou, Renhuai, China; 3 Department of Cardiology, Affiliated Hospital of Zunyi Medical University, Guizhou, Zunyi, China

**Keywords:** ferroptosis, ferroptosis suppressor protein 1, organ injury, sepsis, STING signaling pathway, therapeutic targets

## Abstract

Sepsis, defined as life-threatening organ dysfunction caused by a dysregulated host response to infection, remains a global health challenge characterized by high mortality and limited therapeutic options. Recent evidence identifies ferroptosis—an iron-dependent form of regulated cell death driven by the lethal accumulation of lipid peroxides—as a pivotal mechanism underlying sepsis-induced organ damage. Ferroptosis Suppressor Protein 1 (FSP1) has emerged as a critical endogenous inhibitor of ferroptosis that operates independently of the canonical GPX4/glutathione system. This review provides a comprehensive overview of recent research on FSP1 in the context of sepsis-associated organ injury. We detail the multifaceted molecular mechanisms through which FSP1 inhibits ferroptosis, including the canonical FSP1-CoQ_10_-NAD(P)H axis, the non-canonical vitamin K cycle, ESCRT-III-mediated membrane repair, and the newly characterized vitamin B2-dependent metabolic stability pathway. Furthermore, we highlight the newly discovered *STING-FSP1* signaling axis, where *cGAS-STING* activation transcriptionally represses FSP1, thereby driving endothelial ferroptosis and vascular leakage during sepsis. Additionally, potentially conserved pathways extrapolated from other acute injury models (e.g., ALKBH5, CD36, and SENP3) are discussed to propose novel research directions. The organ-specific roles and protective mechanisms of FSP1 in sepsis-induced injuries of the lungs, heart, liver, kidneys, and brain are systematically summarized. Finally, we evaluate the prospects and challenges of targeting FSP1-related pathways, such as Nrf2 activation and *STING* inhibition, as potential therapeutic strategies for sepsis. Overall, This review aims to provide new insights into the “inflammation-cell death” cascade in sepsis and offer a theoretical foundation for developing FSP1-targeted interventions to suppress ferroptosis.

## 1 Introduction

Sepsis is characterized by a dysregulated host response to infection, presenting a complex pathophysiology driven by immune hyperactivation, endothelial barrier disruption, and coagulopathy (1, 2). This condition frequently progresses to multiple organ dysfunction syndrome (MODS) and represents a primary cause of mortality in critically ill patients (3, 4). Epidemiological assessments from 2021 indicate approximately 166 million sepsis cases globally, contributing to over 21.4 million deaths (5, 6). Despite its high clinical burden, effective treatment options remain limited, underscoring the need for a deeper understanding of the molecular mechanisms underlying cellular and organ damage.

Ferroptosis is an iron-dependent, regulated form of cell death characterized by lipid peroxidation, and it is a key driver of sepsis-induced organ damage (7). Inflammation and oxidative stress caused by sepsis disrupt iron homeostasis and induce ferroptosis (8). Synthesized by the liver to limit extracellular iron availability to pathogens during infection, hepcidin restricts cellular iron efflux by triggering the degradation of the iron exporter ferroportin 1 (FPN1), which subsequently lowers serum iron levels (9–11). However, this host-protective mechanism concomitantly sequesters iron inside cells, expanding the intracellular labile iron pool (LIP) and accelerating lipid peroxidation via the Fenton reaction. Furthermore, the subsequent rupture of ferroptotic cells releases intracellular iron, which can paradoxically exacerbate local iron overload and support bacterial growth (12, 13). Consequently, while host-protective sequestration initially lowers serum iron levels, clinical data reveal that elevated serum iron at later stages of sepsis correlates with poor prognosis and increased mortality (9). This clinical discrepancy reflects the dynamic, temporal translocation of iron—shifting from protective intracellular sequestration to pathological extracellular release following widespread cell lysis.

Among the mechanisms of ferroptosis resistance, FSP1 is a key protein that operates independently of the classical GPX4 system (14). Specifically, FSP1 safeguards cellular integrity against ferroptotic ruin through multiple molecular arms, including the classical coenzyme Q_10_ (CoQ_10_) reduction, the non-canonical vitamin K cycle, the ESCRT-III-dependent physical membrane repair, and the recently unveiled vitamin B2/FAD-dependent homeostatic stability pathway (15–19). Notably, recent studies indicate that *cGAS-STING* pathway activation suppresses FSP1 expression at the transcriptional level, which impairs cell viability, drives endothelial ferroptosis, and increases vascular permeability (20). This underscores the central role of the *cGAS-STING-FSP1* signaling axis in sepsis-induced organ damage.

While the regulatory network of FSP1 has been extensively explored in oncology, its role in acute systemic inflammatory conditions like sepsis remains in the early stages of investigation. Consequently, its precise contributions to various organ injuries and its viability as a therapeutic target have not yet been systematically reviewed. This paper systematically discusses the molecular functions of FSP1, its upstream regulatory networks in sepsis, its specific roles across multiple organ injuries, and its potential as a clinical target to provide a framework for novel intervention strategies.

## 2 Ferroptosis in sepsis

### 2.1 Iron metabolism imbalance

Systemic dysregulation of iron metabolism serves as a primary initiator of ferroptosis during sepsis. Lipopolysaccharide (LPS), a major pathogen-associated molecular pattern (PAMP) from Gram-negative bacteria, binds to Toll-like receptor 4 (TLR4) to initiate host immune responses (21). Upon binding PAMPs or endogenous damage-associated molecular patterns (DAMPs), TLR4 activates NF-κB and mitogen-activated protein kinase (MAPK) pathways via a MyD88-dependent mechanism, promoting the transcription of pro-inflammatory cytokines such as TNF-α and IL-6 (22, 23).

Subsequent signaling involves IL-6 binding to its surface receptor (IL-6R) on hepatocytes to form an IL-6/IL-6R complex. This complex recruits the signaling protein gp130, forming a trimeric assembly that activates constitutively bound Janus kinase 2 (JAK2). Activated JAK2 phosphorylates tyrosine residues on the cytoplasmic domain of gp130, creating docking sites for signal transducer and activator of transcription 3 (STAT3). Once recruited and phosphorylated, STAT3 dimerizes and translocates into the nucleus, where it binds the promoter region of the HAMP gene to drive hepcidin transcription (22, 24). Elevated hepcidin levels induce the internalization and degradation of ferroportin 1 (FPN1), the sole known cellular iron exporter, thereby blocking iron efflux (25, 26). Concurrently, inflammation enhances transferrin receptor 1 (TfR1)-mediated iron uptake and accelerates ferritinophagy—the autophagic degradation of ferritin (27–29). These events collectively expand the intracellular labile iron pool (LIP), demonstrating that the IL-6/JAK2/STAT3/hepcidin axis is a central driver of sepsis-associated iron overload.

### 2.2 Lipid metabolism

In the oxidative microenvironment of sepsis, excess Fe^2+^ within the LIP converts hydrogen peroxide (H_2_O_2_) into highly reactive hydroxyl radicals (·OH) via the Fenton reaction, directly attacking cell membranes (26, 30). Concurrently, inflammatory signaling upregulates long-chain acyl-CoA synthetase 4 (ACSL4) and lysophosphatidylcholine acyltransferase 3 (LPCAT3) (31). ACSL4 catalyzes the activation of long-chain polyunsaturated fatty acids (PUFAs), such as arachidonic acid, into PUFA-CoA, which are subsequently esterified by LPCAT3 into membrane phospholipids (PUFA-PLs) (32). These PUFA-rich phospholipids undergo oxidation by lipoxygenases (LOXs), generating substantial lipid hydroperoxides that cause structural membrane damage, cellular dysfunction, and subsequent ferroptosis (33).

### 2.3 Classic antioxidant system

To counteract lipid peroxidation, cells rely on endogenous antioxidant systems. A central component is the System Xc^–^ transporter—a heterodimer of SLC3A2 and SLC7A11—which facilitates the stoichiometric exchange of extracellular cystine for intracellular glutamate. Upon entry, cystine is rapidly reduced to cysteine, the rate-limiting precursor for glutathione (GSH) biosynthesis (34–36). GSH serves as an essential cofactor for glutathione peroxidase 4 (GPX4), the primary enzyme that reduces lipid hydroperoxides to non-toxic lipid alcohols (37, 38). Preclinical studies have also confirmed that GPX4 and GSH levels are significantly reduced in sepsis (39). Therefore, functional failure of the System Xc^–^-GSH-GPX4 axis leads to impaired cellular capacity to repair lipid peroxidation damage.

**Figure 1:**
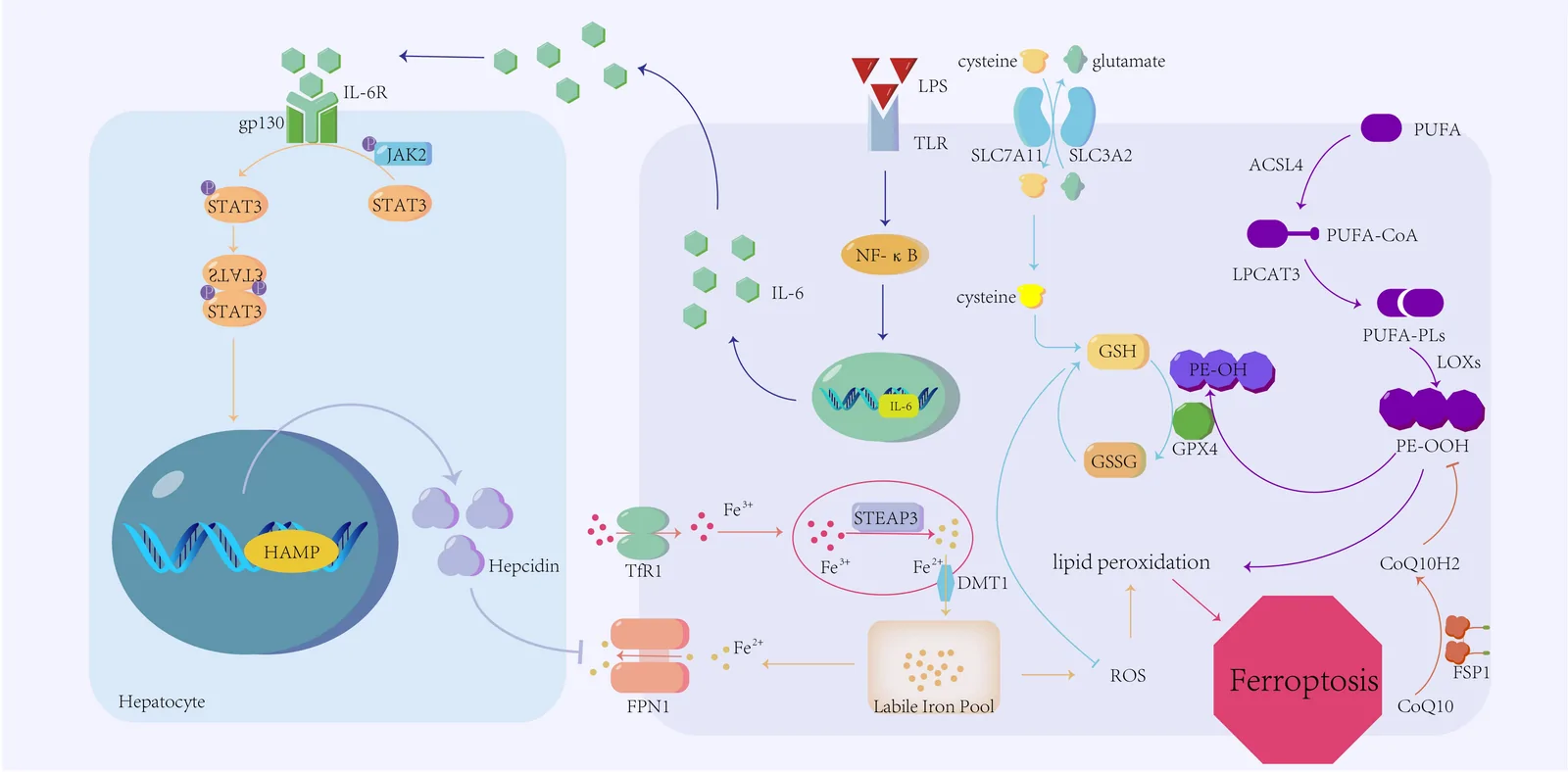
Integrated molecular landscape of ferroptosis during sepsis. Sepsis-induced systemic inflammation triggers iron dyshomeostasis and suppresses antioxidant defense systems (including the GPX4 and FSP1 pathways), collectively driving lipid peroxidation and subsequent multi-organ damage. LPS, Lipopolysaccharide; TLR, Toll-like receptor; NF-κ B, Nuclear factor-kappa B; IL-6, Interleukin-6; IL-6R, IL-6 receptor; JAK2, Janus kinase 2; STAT3, Signal transducer and activator of transcription 3; HAMP, Hepcidin antimicrobial peptide; TfR1, Transferrin receptor 1; FPN1, Ferroportin 1; STEAP3, Six-transmembrane epithelial antigen of prostate 3; DMT1, Divalent metal transporter 1; SLC7A11, Cystine/glutamate antiporter XCT; GSH, Glutathione; GSSG, Glutathione disulfide; GPX4, Glutathione peroxidase 4; ACSL4, Acyl-CoA synthetase long-chain family member 4; LPCAT3, Lysophosphatidylcholine acyltransferase 3; PUFA, Polyunsaturated fatty acid; LOXS, Lipoxygenases; PE-OOH, Phosphatidylethanolamine hydroperoxides; FSP1, Ferroptosis suppressor protein 1; CoQ10, Coenzyme Q10; ROS, Reactive oxygen species.

In brief, sepsis-induced multiorgan dysfunction involves a coordinated cascade spanning IL-6-driven iron overload, ACSL4-mediated lipid peroxidation, and the functional collapse of the canonical System Xc^–^-GSH-GPX4 pathway (**Figure 1**). Although this GPX4 axis has long been considered the primary cellular defense, emerging evidence identifies FSP1 as an independent parallel safeguard in this hyperinflammatory landscape.

## 3 Structure and function of FSP1

### 3.1 Structure and cellular localization

**Figure 2:**
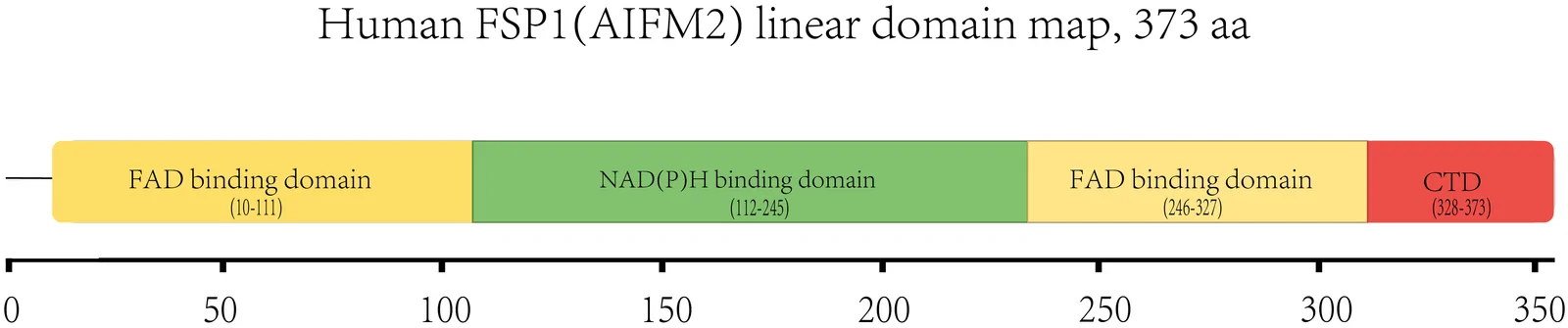
Linear domain architecture of human FSP1 (AIFM2). FAD, flavin adenine dinucleotide; NAD(P)H, nicotinamide adenine dinucleotide phosphate; CTD, C-terminal domain.

FSP1 was initially recognized as a key regulator of apoptosis. Wu et al. first identified the FSP1 gene as a novel p53-responsive gene involved in tumor regulation, subsequently naming it p53-responsive gene 3 (40). Due to structural homology with mitochondrial apoptosis-inducing factor (AIF), it was later designated as Apoptosis-Inducing Factor Mitochondria-Associated 2 (AIFM2) (41). In 2019, a landmark genome-wide CRISPR-Cas9 screen identified FSP1 as a potent ferroptosis inhibitor functioning independently of the canonical GPX4 pathway; consequently, the gene was renamed FSP1 to reflect its central anti-ferroptotic role (15). The human FSP1 gene is mapped to chromosome 10q21.3–q22.1 and encodes a 373-amino acid polypeptide with a theoretical molecular weight of approximately 40.5 kDa (40). FSP1 predominantly localizes to the plasma membrane, lipid droplets, and other endomembrane structures (41–43). Structurally, FSP1 consists of two tandem Rossmann fold domains (RFDs) and a C-terminal domain (CTD); the RFDs are responsible for binding the cofactors 6-hydroxyflavin adenine dinucleotide (FAD) and nicotinamide adenine dinucleotide (NAD(P)H), respectively, while the CTD mediates substrate binding and membrane anchoring (44). Notably, FSP1 contains an N-terminal myristoylation motif, which is indispensable for its membrane recruitment (42). Crystal structure analysis has further delineated its functional architecture: the 6-OH-FAD-binding domain (residues 10–111 and 246–327), the NAD(P)H-binding domain (residues 112–245), and the C-terminal membrane-anchoring domain (residues 328–373) (45) (**Figure 2**). Recent breakthroughs have revealed that FSP1 possesses an intrinsically disordered region (IDR) capable of driving liquid-liquid phase separation (LLPS). This allows FSP1 to form functional condensates at damaged membrane sites, facilitating efficient local scavenging of lipid peroxyl radicals and accelerating membrane repair (46). Functionally, FSP1, which is localized on the surface of lipid droplets, uses NADH to reduce CoQ_10_ to its antioxidant form, CoQ_10_H_2_, thereby directly scavenging lipid free radicals generated by neutral lipids (such as triglycerides and cholesterol esters) within the lipid droplets and preventing the peroxidation of neutral lipids and ferroptosis (43). Additionally, the lipid droplet surface protein HSD17B11 physically interacts with FSP1, maintaining its stability and abundance on these lipid structures (47). In terms of tissue distribution, FSP1 expression is most abundant in lipid-rich environments, such as adipose tissue and the liver, and is significantly expressed in the kidneys, lungs, and brain (43). This distribution pattern suggests that FSP1 acts as a critical protective hub in tissues characterized by active lipid metabolism or high susceptibility to oxidative stress.

### 3.2 Core antioxidant and repair mechanisms

#### 3.2.1 The FSP1- CoQ_10_-NAD(P)H axis

FSP1 is targeted to and localized on the plasma membrane via N-terminal myristoylation. Utilizing NAD(P)H as an electron donor, it transfers electrons via its cofactor FAD to reduce CoQ_10_ on the membrane to CoQ_10_H_2_. The resulting CoQ_10_H_2_ can directly scavenge and neutralize lipid peroxyl radicals in the plasma membrane, thereby terminating the chain reaction of lipid peroxidation and playing a key role in preventing ferroptosis (15). Ferroptotic stress can also activate AMPK, promoting the translocation of aldehyde dehydrogenase 7A1 (ALDH7A1) to the plasma membrane. ALDH7A1 clears reactive aldehydes and locally generates NADH to supply electrons to FSP1, establishing an ALDH7A1-NADH-FSP1 metabolic axis that optimizes antioxidant efficiency (48). Functionally, when FSP1 is absent, supplementation with vitamin E partially restores cells’ resistance to ferroptosis, suggesting that vitamin E can, to some extent, compensate for the function of FSP1 (49–51).

#### 3.2.2 FSP1-vitamin K-NAD(P)H pathway

As an active vitamin K reductase, FSP1 catalyzes a non-canonical vitamin K cycle to reduce vitamin K into its hydroquinone form (VKH2) at the expense of NAD(P)H, where the resulting VKH2 acts as a potent antioxidant to directly scavenge lipid free radicals, thereby effectively preventing lipid peroxidation and inhibiting the onset of ferroptosis (16, 52). Under GPX4 inactivation, specific forms of vitamin K, including phylloquinone (VK1), menatetrenone-4 (MK-4), and menadione, inhibit ferroptosis through this FSP1-dependent pathway (52). Furthermore, research has shown that vitamin K2 may upregulate FSP1 expression by activating the Nrf2 signaling pathway in disease models such as bone metabolism, thereby exerting an inhibitory effect on ferroptosis; this provides a new perspective on the regulation of the FSP1- vitamin K pathway (53).

#### 3.2.3 FSP1-ESCRT-III de-SUMOylation

FSP1 directly repairs plasma membrane damage by recruiting and activating the endosomal sorting complex required for transport (ESCRT)-III machinery, thereby inhibiting ferroptosis. During ferroptosis, lipid peroxidation leads to the formation of nanoscale pores in the plasma membrane and triggers a sustained influx of calcium ions (Ca^2+^), which serves as a signal to activate this ESCRT-III-dependent repair mechanism (54). FSP1 translocates to these damaged membrane sites and recruits ESCRT-III core subunits, such as CHMP5 and CHMP6, initiating a budding mechanism that sheds damaged membrane segments. This pathway preserves plasma membrane integrity, delays lysis, and modulates the release of immunogenic DAMPs (17, 18, 54).

#### 3.2.4 The FSP1-vitamin B2 axis

Within cells, vitamin B2 (riboflavin) is converted into flavin adenine dinucleotide (FAD) through a series of enzymatic reactions catalyzed by riboflavin kinase (RFK) and FAD synthase (FLAD1). FAD binding is essential for maintaining FSP1 structural stability and catalytic activity. In the absence of FAD binding, FSP1 is recognized by the E3 ubiquitin ligase RNF8 and subsequently undergoes degradation by the 26S proteasome via K48-linked polyubiquitination (19, 55).Therefore, maintaining adequate intracellular levels of vitamin B2 and FAD is a key upstream determinant of FSP1 protein homeostasis, suggesting that targeting the FSP1/vitamin B2 pathway may offer a novel strategy for enhancing anti-ferroptotic defenses.

### 3.3 Crosstalk with parallel ferroptosis pathways

#### 3.3.1 The system Xc^–^-GSH-GPX4 axis

Systemic metabolic stress and oxidative conditions during sepsis often deplete cysteine and GSH, decreasing GPX4 expression or activity (39). When this canonical axis is impaired, cell survival becomes dependent on FSP1. Studies have demonstrated a robust functional redundancy and reciprocal compensation between the FSP1 and GPX4 systems: for instance, pharmacological or genetic inhibition of GPX4 often triggers the up-regulation of FSP1 to sustain cell viability; conversely, FSP1 deficiency may induce a compensatory increase in GPX4 activity (15, 51).

#### 3.3.2 DHODH-CoQ_10_ pathway

DHODH is an enzyme encoded by the DHODH gene on human chromosome 16. The protein is primarily localized to the inner mitochondrial membrane (IMM), where it catalyzes the oxidation of dihydroorotate to orotate while simultaneously reducing mitochondrial CoQ_10_ to ubiquinol CoQ_10_H_2_), a process essential for pyrimidine biosynthesis and DNA replication (56, 57). While DHODH and FSP1 both utilize CoQ_10_ reduction to suppress lipid peroxidation, they operate in distinct compartments (mitochondria versus plasma membrane) (58). Complex crosstalk exists between these systems; for instance, the pro-ferroptotic effect of the DHODH inhibitor Brequinar is attenuated in FSP1-knockout lines despite normal DHODH expression, indicating a functional hierarchy or spatial compensation between mitochondrial and plasma membrane antioxidant networks.

**Figure 3:**
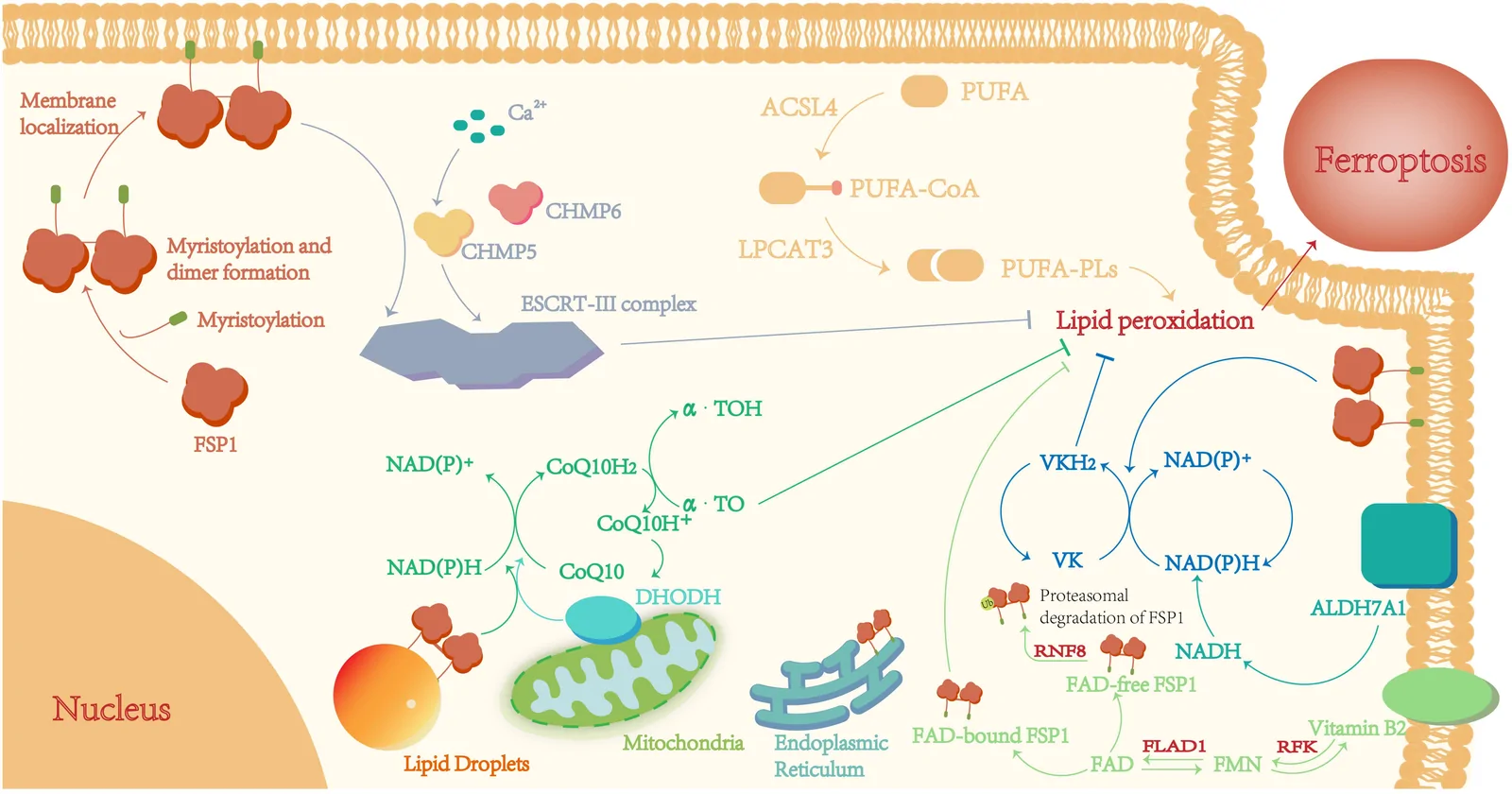
The multifaceted regulatory landscape and antioxidant mechanisms of FSP1. FSP1 undergoes N-myristoylation and dimerization for plasma membrane recruitment, where it suppresses ferroptosis through the CoQ10 and Vitamin K reduction axes. Its functional integrity is orchestrated by the ESCRT-III-mediated membrane repair, FAD-dependent protein stability, and sophisticated metabolic pathways. FSP1, Ferroptosis suppressor protein 1; ESCRT-III, Endosomal sorting complex required for transport-III; CHMP5/6, Charged multivesicular body protein 5/6; ACSL4, Acyl-CoA synthetase long-chain family member 4; LPCAT3, Lysophosphatidylcholine acyltransferase 3; PUFA-PLs, Polyunsaturated fatty acid-containing phospholipids; CoQ10, Coenzyme Q10; VK, Vitamin K; a TOH, a -tocopherol; DHODH, Dihydroorotate dehydrogenase; RNF8, Ring finger protein 8; FLAD1, FAD synthase 1; RFK, Riboflavin kinase; ALDH7A1, Aldehyde dehydrogenase 7 family member A1; FAD, Flavin adenine dinucleotide; FMN, Flavin mononucleotide.

Research has highlighted a complex interplay between these two systems. For instance, the DHODH inhibitor Brequinar has been shown to synergistically induce ferroptosis in cells with low GPX4 expression. Notably, the pro-ferroptotic effect of Brequinar is significantly attenuated in FSP1-knockout cells, even when DHODH expression remains normal (59). This suggests a sophisticated functional hierarchy or compensatory crosstalk between the mitochondrial DHODH system and the plasma membrane-localized FSP1 system. Consequently, DHODH may operate as a key partner or downstream effector in the broader FSP1-mediated antioxidant network, working in tandem to maintain cellular redox homeostasis under diverse stress conditions (**Figure 3**).

## 4 Regulation of FSP1 expression

### 4.1 Transcriptional regulation

#### 4.1.1 The Nrf2-FSP1 axis

The KEAP1-Nrf2 system serves as the master regulatory network for the cellular antioxidant response and plays a pivotal role in shielding cells from lipid peroxidation and ferroptotic damage. Under physiological homeostasis, the transcription factor Nrf2 is sequestered in the cytoplasm by its adaptor protein, KEAP1. KEAP1 functions as a substrate receptor for the Cullin 3-based E3 ubiquitin ligase complex, mediating the continuous ubiquitination and subsequent proteasomal degradation of Nrf2, thereby maintaining it at low basal levels (60, 61). In the context of sepsis, endotoxins and the excessive generation of ROS can modify key cysteine residues (notably Cys151) on KEAP1. This modification triggers a conformational shift in KEAP1, disrupting its E3 ligase activity and effectively inhibiting Nrf2 degradation (62). Consequently, stabilized Nrf2 undergoes nuclear translocation and forms a heterodimer with small Maf (sMAF) proteins. This complex specifically recognizes and binds to the antioxidant response elements (ARE) located in the promoter regions of target genes, including FSP1, thereby initiating its transcription (63–65). Therefore, the activation of the Nrf2-FSP1 signaling axis represents a critical endogenous compensatory mechanism for upregulating FSP1 and enhancing cellular resistance to ferroptosis during septic challenges.

#### 4.1.2 The *cGAS-STING-FSP1* axis

The cGAS-*STING* pathway negatively regulates FSP1 expression during sepsis. Sepsis-induced IL-6 elevation promotes the release of mitochondrial DNA (mtDNA) into the cytoplasm, where it binds to cyclic GMP-AMP synthase (cGAS) and activates the stimulator of interferon genes (*STING*) cascade (20, 66–68). *STING* activation subsequently downregulates FSP1 in endothelial cells, facilitating endothelial ferroptosis and vascular barrier disruption (69). Upstream, enhanced fatty acid synthesis drives voltage-dependent anion channel 1 (VDAC1) oligomerization by suppressing its ETS1-mediated ubiquitination. This increase in mitochondrial membrane permeability accelerates mtDNA leakage, amplifying *cGAS-STING* signaling. Consistently, blocking fatty acid synthesis alleviates sepsis-induced endothelial injury, highlighting the therapeutic relevance of this metabolic-immune axis (70). Additionally, *cGAS-STING* signaling promotes ferritinophagy and NLRP3 inflammasome activation, coupling ferroptosis with systemic inflammation (20, 71). Intervening in the FASN/ETS1/*STING* axis to restore *FSP1* expression represents a potential strategy to mitigate septic endothelial failure.

#### 4.1.3 The SIRT5-HOXA5-FSP1 axis

In a sepsis-induced acute lung injury (ALI) model, the expression of the transcription factor HOXA5 and its downstream target FSP1 is significantly downregulated in injured lung tissues. Mechanistically, the desuccinylase SIRT5 mediates the desuccinylation of the HOXA5 protein, which markedly enhances its binding affinity to the FSP1 promoter, thereby upregulating FSP1 expression at the transcriptional level (72). Suppression of SIRT5 activity within the septic microenvironment leads to HOXA5 inactivation, contributing to the decline of FSP1-mediated defenses.

#### 4.1.4 The IRF1-FSP1 axis

IRF1, a canonical downstream effector of interferon (IFN) signaling, plays a pivotal role in driving septic inflammation. Upon septic challenges, the activation of pattern recognition receptors by PAMPs or DAMPs triggers the phosphorylation, homodimerization, and nuclear translocation of STAT1, which directly binds to the IRF1 promoter to upregulate its transcription (73). Consequently, IRF1 expression is markedly elevated during sepsis progression, and its genetic ablation or pharmacological inhibition significantly alleviates sepsis associated inflammatory lung injury (74). This underscores the critical role of IRF1 as a pathogenic driver during sepsis. Although direct evidence in native sepsis models is currently lacking, mechanistic insights from viral infection (such as influenza A virus) models demonstrate that IRF1 directly binds to the FSP1 promoter as a transcriptional repressor, downregulating FSP1 expression and facilitating ferroptosis (75). Given that the interferon signaling pathway is widely activated during the systemic inflammatory response in sepsis, whether and how IRF1 participates in the transcriptional regulation of FSP1 under sepsis conditions is an important scientific question that urgently needs to be addressed. Elucidating this mechanism will provide a new perspective on the interaction between the immune response and the ferroptosis defense system.

### 4.2 Post-transcriptional regulation

M^6^A RNA methylation, a pivotal determinant of mRNA processing and decay, represents an emerging frontier in understanding septic organ dysfunction. In a model of polymicrobial sepsis induced by cecal ligation and puncture (CLP), ALKBH5 deficiency significantly increased mortality in mice. Specifically, Alkbh5-deficient CLP mice exhibited impaired neutrophil migration, leading to increased bacterial loads in the peritoneal cavity and bloodstream, accompanied by excessive production of pro-inflammatory cytokines (76). This suggests that ALKBH5 may have a protective effect in sepsis. However, it remains unclear whether ALKBH5 mediates this protective effect through specific downstream targets. Although there is no direct evidence elucidating the role of the ALKBH5-FSP1 axis in sepsis, key insights from myocardial ischemia/reperfusion (I/R) models highlight its potential pathological relevance. Mechanistically, ALKBH5 directly binds to m6A modification sites on FSP1 mRNA and demethylate them, thereby preventing transcript degradation and maintaining FSP1 expression, which protects cardiomyocytes from damage caused by lipid peroxidation and ferroptosis (77). In addition, several oncology studies have confirmed the presence of abundant m^6^A modification sites on FSP1 mRNA (78–80), This further suggests that FSP1 expression may be broadly subject to m^6^A - dependent post-transcriptional regulation. Given that oxidative stress and ferroptosis are pivotal pathological phenomena shared by both sepsis and I/R injury, further investigation is highly warranted to systematically validate whether this ALKBH5/m^6^A/FSP1 regulatory axis operates in native sepsis models.

### 4.3 Post-translational modifications

#### 4.3.1 CD36-mediated ubiquitination and degradation

Post-translational modifications regulate the stability of the FSP1 protein, which directly affects cellular survival under infectious stress. In sepsis, elevated CD36 levels have been identified as an independent risk factor for immunosuppression and patient mortality (81). Furthermore, its level is positively correlated with the severity of sepsis-related injury (81–83). However, the underlying mechanism remains unclear. Studies using a cisplatin-induced non-septic acute kidney injury (AKI) model have provided clues regarding the mechanism: CD36 directly bind to FSP1 and recruit an E3 ubiquitin ligase, promoting the ubiquitination and degradation of FSP1, thereby impairing the resistance of renal tubular epithelial cells to ferroptosis (84). Based on this, we hypothesize that the CD36-FSP1 axis may drive ferroptosis-mediated kidney injury in sepsis; this hypothesis requires further validation in sepsis models.

#### 4.3.2 SENP3-mediated De-SUMOylation

**Figure 4:**
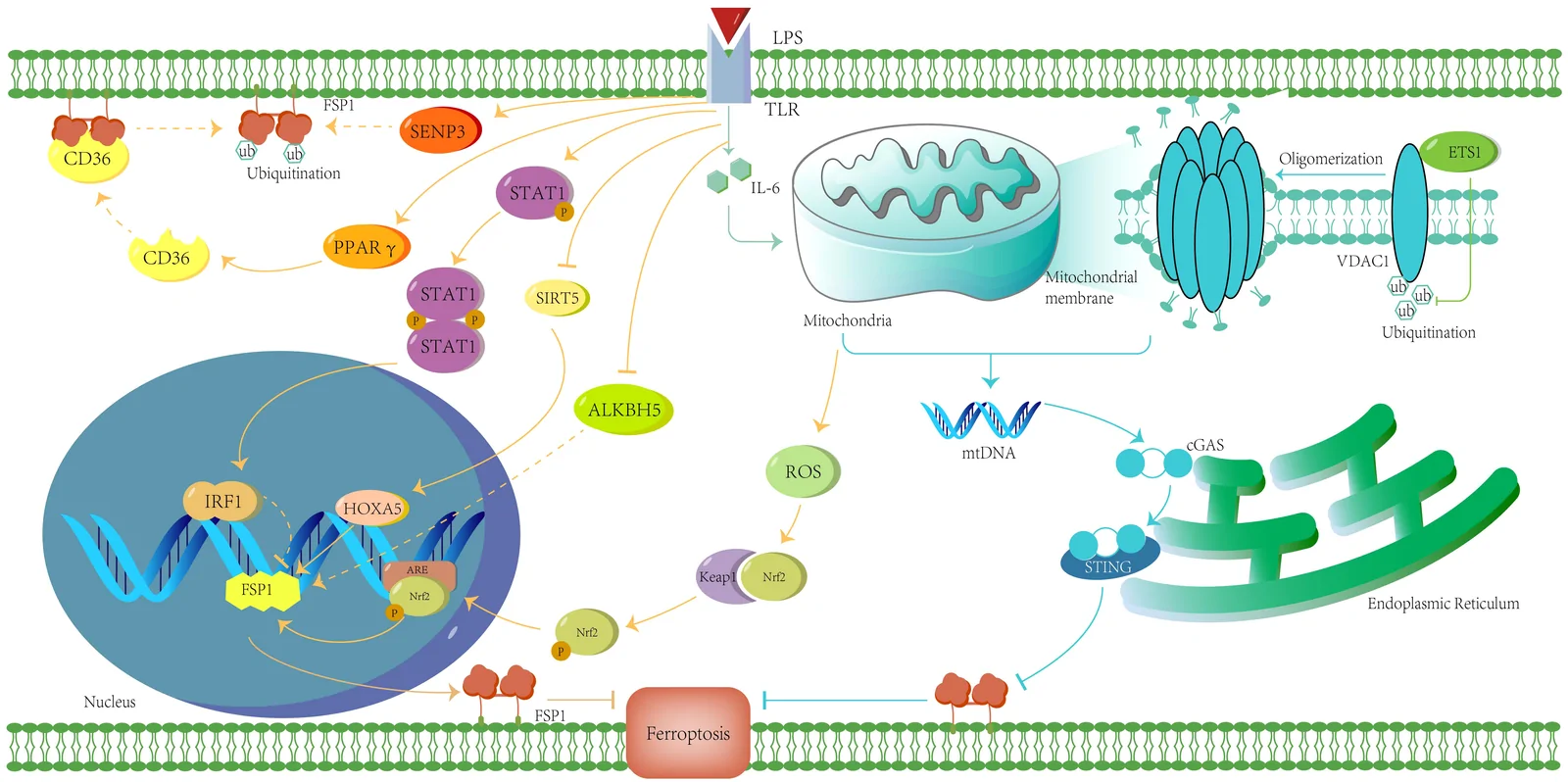
Sepsis triggers complex inflammatory and oxidative signals that regulate FSP1 expression and stability at multiple levels. Solid lines indicate established pathways verified in sepsis models, whereas dashed lines represent potential regulatory mechanisms extrapolated from other injury models that warrant direct validation in native sepsis. Transcriptional Regulation: ROS stabilizes Nrf2 to activate FSP1 transcription, and the SIRT5-HOXA5 axis upregulates FSP1. Conversely, the CGAS-STING axis and the IRF1 pathway act as transcriptional repressors to downregulate FSP1.Post-transcriptional Regulation: The m6A demethylase ALKBH5 removes m6A modifications from FSP1 mRNA, preventing transcript degradation. Post-translational Modification: CD36 recruits E3 ligases to facilitate ubiquitin-dependent FSP1 degradation. Concurrently, the de-SUMOylase SENP3 removes SUMO modifications from FSP1, undermining its stability and accelerating its proteasomal clearance. ALKBH5, Alkylation repair homolog 5; ARE, Antioxidant response element; cGAS, Cyclic GMP-AMP synthase; ETS1, Protein C-ets-1; FSP1, Ferroptosis suppressor protein 1; HOXA5, Homeobox A5; IRF1, Interferon regulatory factor 1; Keap1, Kelch-like ECH-associated protein 1; LPS, Lipopolysaccharide; m6A, N6-methyladenosine; mtDNA, Mitochondrial DNA; Nrf2, Nuclear factor erythroid 2-related factor 2; PPAR y, Peroxisome proliferator-activated receptor gamma; ROS, Reactive oxygen species; SENP3, Sentrin-specific protease 3; SIRT5, Sirtuin 5; STAT1, Signal transducer and activator of transcription 1; STING, Stimulator of interferon genes; TLR, Toll-like receptor; VDAC1, Voltage-dependent anion channel 1.

Post-translational modifications also regulate FSP1 protein stability through dynamic SUMOylation. During sepsis, high concentrations of ROS activate the deSUMOylase SENP3 (85, 86), but whether SENP3 directly contributes to septic organ injury by targeting FSP1 remains unknown. Mechanistic evidence from basal macrophage studies shows that SENP3 binds to the K162 site of FSP1 to catalyze its de-SUMOylation. This modification reduces FSP1 stability and accelerates its ubiquitin-proteasome-dependent degradation, thereby compromising the anti-ferroptotic capacity of macrophages (87). Given the severe oxidative stress environment in sepsis, we hypothesize that the ROS-SENP3-FSP1 axis may serve as a potential pathological cascade driving ferroptosis-mediated organ damage, though this extrapolation requires direct validation in native sepsis models (**Figure 4**).

## 5 The role of FSP1-mediated ferroptosis suppression in sepsis-induced multiorgan injury

### 5.1 Acute lung injury/ARDS

Acute lung injury (ALI) and acute respiratory distress syndrome (ARDS) are severe and frequent complications of sepsis, characterized by the disruption of the alveolar-capillary barrier due to the massive death of alveolar epithelial cells. This process leads to diffuse alveolar damage, refractory hypoxemia, and respiratory failure (88, 89). In ALI/ARDS models, lung tissues exhibit hallmark features of ferroptosis, including shrunken mitochondria with reduced cristae, iron overload, and the accumulation of lipid peroxides (90, 91).

FSP1 expression in the septic lung is regulated by transcriptional and metabolic inputs. At the transcriptional level, the desuccinylase SIRT5 mediates the desuccinylation of HOXA5, which enhances the binding affinity of the transcription factor HOXA5 to the FSP1 promoter, thereby upregulating its expression. This SIRT5/HOXA5/FSP1 axis effectively inhibits ferroptosis in lipopolysaccharide (LPS)-induced lung epithelial cells (e.g., MLE-12 cells), offering a novel molecular target for sepsis management (72). At the metabolic level, inhibitors targeting Heme Oxygenase-1 (Hmox1) can activate the FSP1/CoQ_10_/NADPH pathway while simultaneously enhancing protective autophagy, synergistically suppressing epithelial ferroptosis and alleviating lung injury in septic mice (92). Together, these findings reveal that FSP1 expression in the septic lung is orchestrated by a complex interplay of epigenetic, transcriptional, and metabolic signals. Targeting FSP1 and its regulatory networks, such as the SIRT5/HOXA5 axis, to inhibit alveolar epithelial ferroptosis represents a highly promising strategy for preserving the alveolar-capillary barrier and improving the prognosis of sepsis-induced ALI/ARDS.

### 5.2 Sepsis-associated acute liver injury

During sepsis, hepatic iron homeostasis and metabolic function are disrupted by inflammatory and oxidative stress. Elevated IL-6 activates the JAK2-STAT3 signaling cascade to upregulate hepcidin, which induces FPN1 internalization and degradation, blocking iron efflux and causing iron accumulation in hepatocytes (93–96). This iron overload drives lipid peroxidation via the Fenton reaction. Septic liver tissues demonstrate elevated iron content, high malondialdehyde (MDA) levels, and suppressed GPX4 expression, alongside mitochondrial shrinkage and loss of cristae (97, 98). Upregulating FSP1 can alleviate this damage. For example, tetramethylpyrazine (TMP) and mitochondrial aldehyde dehydrogenase 2 (ALDH2) protect hepatocytes by increasing FSP1 expression (99, 100). Therefore, targeting the FSP1 pathway to restore lipid antioxidant capacity represents a highly promising therapeutic strategy for mitigating sepsis-associated acute liver injury.

### 5.3 Sepsis-associated acute kidney injury

Sepsis-associated acute kidney injury (SA-AKI) is associated with high metabolic activity and mitochondrial density in renal tubular cells, making them sensitive to lipid peroxidation (101–104). Elevated serum ferritin levels in SA-AKI patients correlate with poor clinical outcomes, confirming clinical iron dysregulation (105). Nrf2 serves as an upstream positive regulator of FSP1 in the kidney. Pharmacological agents such as dexmedetomidine (DEX) and andrographolide (AG) reduce renal tubular cell ferroptosis by activating the Nrf2/FSP1 pathway (64, 106). However, this anti-ferroptotic shield may be post-translationally compromised. Based on insights from non-septic AKI models, the transmembrane protein CD36 can promote FSP1 ubiquitination and degradation, thereby exacerbating tubular injury (84). Given that CD36 is an inflammatory mediator upregulated during early sepsis (81), this inflammation-driven expression is hypothesized to weaken the renal antioxidant barrier by accelerating FSP1 clearance. Conversely, natural compounds like ginsenoside Rg1 stabilize FSP1 protein expression, counteracting the ferroptotic process in SA-AKI (107).

### 5.4 Sepsis-induced myocardial dysfunction

Sepsis-induced myocardial dysfunction (SIMD) significantly contributes to mortality in critically ill patients, with ferroptosis operating as a driver of tissue injury (39, 108). Septic myocardial tissues display myofibrillar disarray and mitochondrial shrinkage, accompanied by marked downregulation of FSP1 protein levels (39, 109). FSP1 expression in the heart is modulated by upstream inflammatory signals. Lipocalin-2 (Lcn2), an iron-regulatory protein upregulated in the septic myocardium, exacerbates ferroptosis by antagonizing FSP1 expression (109). Pharmacological interventions can counter this suppression; curcumin nanoparticles (Cur-NPs) activate the Nrf2/FSP1 signaling axis, upregulating FSP1, quenching lipid peroxidation, and preserving cardiac function (110). Restoring FSP1 expression via Lcn2 inhibition or Nrf2 activation represents a potential therapeutic approach for SIMD.

### 5.5 Sepsis-associated encephalopathy

**Figure 5:**
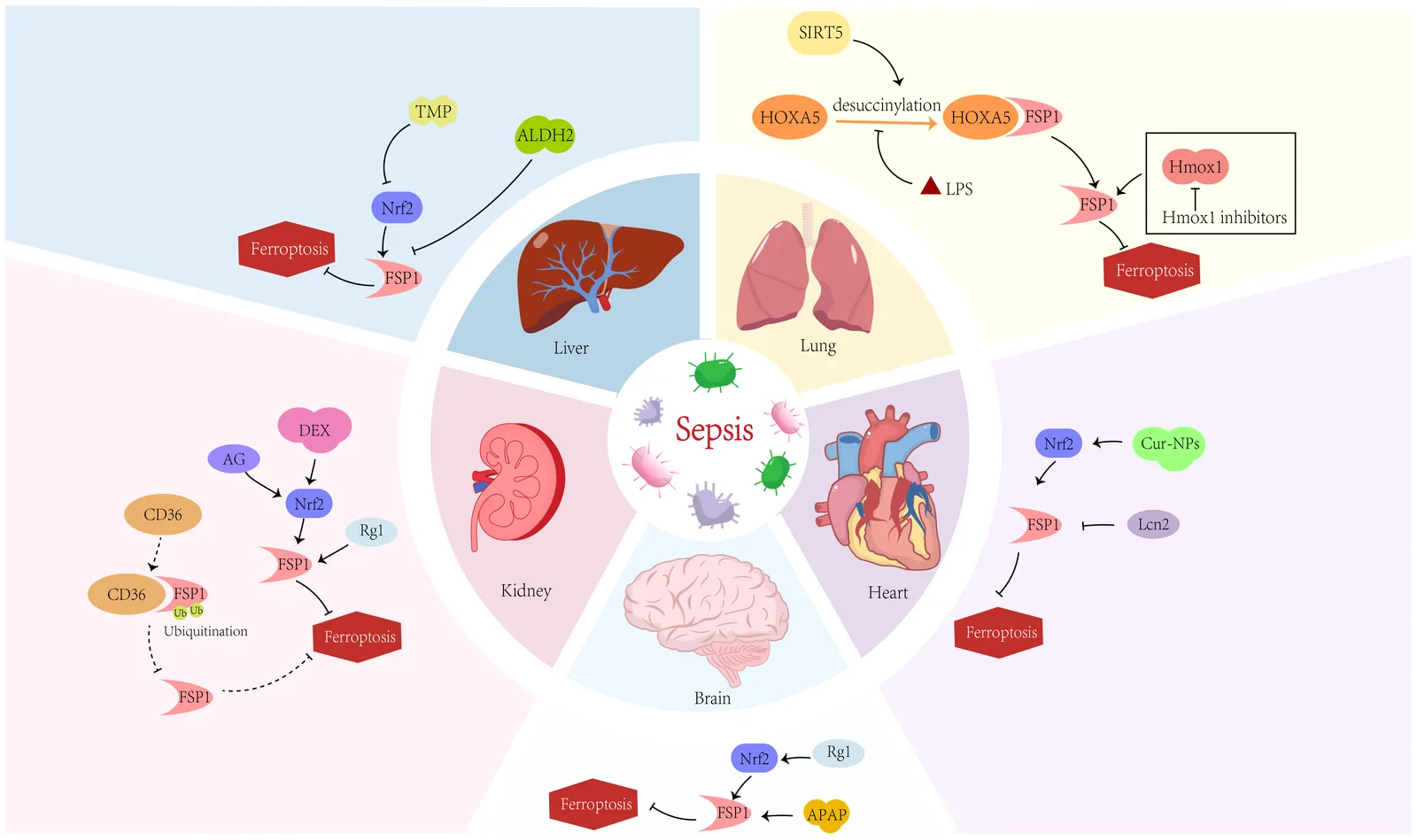
FSP1-mediated ferroptosis suppression in sepsis: regulatory mechanisms and organ damage. FSP1 plays a pivotal role in the pathogenesis of sepsis-induced multi-organ injury, including the lung, liver, kidney, heart, and brain. SIRT5, Sirtuin 5; HOXA5, Homeobox A5; Hmox1, Heme oxygenase 1; LPS, Lipopolysaccharide; ALDH2, Aldehyde dehydrogenase 2; TMP, Tetramethylpyrazine; Nrf2, Nuclear factor erythroid 2-related factor 2; DEX, Dexmedetomidine; AG, Andrographolide; Rg1, Ginsenoside Rg1; CD36, Cluster of differentiation 36; Ub, Ubiquitin; Cur-NPs, Curcumin nanoparticles; Lcn2, Lipocalin-2; APAP, Acetaminophen; ROS, Reactive oxygen species.

Sepsis-associated encephalopathy (SAE) presents as acute or long-term cognitive dysfunction linked to neuronal ferroptosis (111). Iron dyshomeostasis induces abnormal iron accumulation within microglia and neurons, driving lipid peroxidation and neurological injury (112–114). FSP1 provides neuroprotection in SAE. Ginsenoside Rg1 upregulates FSP1 expression via Nrf2 pathway activation, alleviating LPS-induced neuronal ferroptosis and cognitive impairment (115). Additionally, acetaminophen (APAP) administration in sepsis models upregulates hippocampal FSP1 expression, reducing tissue iron content, lipid peroxidation markers (4-HNE), and ROS levels, which mitigates hippocampal injury and improves survival rates (116). These findings position FSP1 as a target for treating sepsis-induced neurological injury (**Figure 5**).

## 6 Therapeutic perspectives and clinical translation

Because FSP1 serves as an anti-ferroptotic safeguard independent of the GPX4 system, it represents a candidate pharmacological target for treating sepsis-associated multiple organ dysfunction syndrome (MODS). Current therapeutic strategies focus primarily on transcriptional modulation and metabolic substrate support. The key pharmacological interventions and target pathways across various organ injuries are summarized in **Table 1**.

**Table 1:** Summary of pharmacological interventions targeting FSP1-mediated ferroptosis supression in sepsis-associated organ injuries.

Affected organ	Intervention agent	Targeted pathway	Main protective effects	References
Lung (*ALI/ARDS*)	*Hmox1 Inhibitors*	FSP1 / CoQ10 / NADPH pathway	Activates FSP1 defense alongside enhancing protective autophagy to suppress lung epithelial cell ferroptosis.	(92)
	*SIRT5*	SIRT5-HOXA5-FSP1 axis	SIRT5 mediates the desuccinylation of HOXA5, enhancing its binding affinity to the *FSP1* promoter; this transcriptional upregulation of FSP1 robustly inhibits epithelial cell ferroptosis.	(72)
Liver (*SA-ALI*)	Tetramethylpyrazine (TMP)	Nrf2 / FSP1 pathway	Inhibits hepatocyte ferroptosis, counteracts iron overload-driven lipid peroxidation, and ameliorates liver injury.	(99)
	ALDH2	ALDH2/FSP1 pathway	Inhibits hepatocyte ferroptosis, counteracts iron overload-driven lipid peroxidation, and ameliorates liver injury	(100)
Kidney (*SA-AKI*)	Dexmedetomidine (DEX)	Nrf2 / FSP1 / CoQ10 pathway	Suppresses renal tubular cell ferroptosis and attenuates sepsis-associated acute kidney injury.	(64)
	Andrographolide (AG)	Nrf2 / FSP1 pathway	Inhibits lipid peroxidation in highly metabolic renal tubular epithelial cells, protecting against tubular injury.	(106)
	Ginsenoside Rg1	FSP1 protein stabilization	Stabilizes FSP1 protein expression to counteract the inflammatory ferroptotic process in SA-AKI.	(107)
Heart (*SIMD*)	Curcumin Nanoparticles (Cur-NPs)	Nrf2/FSP1 signaling axis	Up-regulates FSP1, quenches lipid peroxidation, and mitigates sepsis-induced myocardial dysfunction.	(110)
	Lipocalin-2 (Lcn2)	Lcn2-mediated FSP1 repression	Pathologically suppresses FSP1 expression to exacerbate myocardial ferroptotic damage.	(109)
Brain (*SAE*)	Ginsenoside Rg1	Nrf2 / FSP1 axis	Upregulates FSP1 expression via Nrf2 pathway, alleviating neuronal ferroptosis and acute/long-term cognitive impairment.	(115)
	Acetaminophen (APAP)	FSP1 protein stabilization	Reduces hippocampal iron content, lipid peroxidation markers (4-HNE), and ROS levels, mitigating neurological injury.	(116)
Multi-Organ (*Systemic*)	*sting Inhibitors*	STING / FSP1 pathway (Antagonism)	Reduces oxidative stress and vascular dysfunction.	(69)
	Coenzyme Q10 / MitoQ	FSP1 / CoQ10 substrate support	Alleviates profound systemic metabolic crisis by ensuring substrate availability for FSP1 enzymatic flux, preserving cardiac and renal functions.	(124–126)

### 6.1 Transcriptional modulation strategies

#### 6.1.1 Activation of the Nrf2/FSP1 axis

The transcription factor Nrf2 directly drives FSP1 expression by binding to the antioxidant response elements (AREs) located within its promoter region (117). Preclinical models of sepsis-associated acute kidney injury (SA-AKI) have confirmed that Nrf2 activators, such as andrographolide, significantly suppress lipid peroxidation in renal tubular epithelial cells by upregulating FSP1 (106). Additionally, clinically utilized agents, including dexmedetomidine, have been shown to exert profound organoprotective effects via the Nrf2/FSP1 pathway (64). Nevertheless, leveraging advanced targeted delivery systems to overcome the low bioavailability of these lipophilic compounds remains a primary focus of current translational research.

It is worth noting that clinically leveraging Nrf2/FSP1 activation in sepsis presents a major translational challenge due to its stark therapeutic contradiction with oncology. While upregulating the Nrf2-FSP1 axis protects normal tissue from septic ferroptotic damage, FSP1 and Nrf2 are notoriously overexpressed in various malignancies (106, 118). In cancer cells, FSP1 serves as a key parallel defense mechanism against iron-dependent death, and its inhibition is highly pursued to sensitize tumors to radiotherapy and chemotherapy (118, 119). Consequently, systemic or prolonged clinical activation of Nrf2/FSP1 to treat sepsis carries the inherent risk of promoting oncogenesis, accelerating tumor growth, or inducing therapy resistance in patients with undiagnosed or developing malignancies. This bidirectional therapeutic paradox underscores that future translation of FSP1-activating therapies must rely on transient, organ-targeted, or precision-controlled delivery systems rather than sustained systemic administration.

#### 6.1.2 Antagonism of the *STING/FSP1* pathway

Hyperactivation of the *cGAS-STING* pathway during sepsis represses FSP1 in endothelial cells, contributing to barrier disruption (69). Blockade of this repressive signaling restores FSP1 defenses. For example, the mitochondrial regulator Mdivi-1 rescues FSP1 capacity by mitigating aberrant *STING* signaling, thereby alleviating sepsis-induced hepatic and myocardial injuries (120, 121).

#### 6.1.3 Targeting the SIRT5-HOXA5-FSP1 cascade

In Sepsis-induced acute lung injury (ALI), the expression of the transcription factor HOXA5 and its downstream target FSP1 is characteristically downregulated. Mechanistically, the desuccinylase SIRT5 mediates the desuccinylation of the HOXA5 protein, which markedly enhances its binding affinity to the FSP1 promoter, thereby upregulating FSP1 transcription (72). Given that the septic microenvironment often triggers STAT1-mediated suppression of SIRT5 activity, pharmacological strategies aimed at restoring SIRT5 desuccinylase activity or employing specific SIRT5 activators represent a novel, sophisticated epigenetic-transcriptional approach to rescue FSP1 expression and protect alveolar epithelial cells from ferroptotic collapse.

### 6.2 Metabolic support: exogenous CoQ_10_ supplementation

The anti-ferroptotic activity of FSP1 is fundamentally dependent on its enzymatic capacity to reduce CoQ_10_ to ubiquinol (CoQH_2_), which acts as a potent lipophilic radical scavenger; thus, maintaining an adequate cellular CoQ_10_ pool is a prerequisite for the integrity of the FSP1/CoQ_10_/NAD(P)H pathway (15, 122). Sepsis frequently precipitates a severe metabolic crisis characterized by profound systemic CoQ_10_ depletion (123). Preclinical studies have corroborated that exogenous supplementation with CoQ_10_ or its mitochondria-targeted formulation (MitoQ) effectively ameliorates sepsis-induced myocardial dysfunction and acute kidney injury (124–126). This metabolic priming approach not only directly ensures the substrate availability required for FSP1 enzymatic flux but also offers a favorable clinical safety profile, making it an indispensable component of future multi-modal anti-ferroptotic therapies for sepsis.

### 6.3 Emerging strategies and translational tools

To facilitate the clinical translation of FSP1-targeted interventions, several cutting-edge technological approaches and clinical tools are currently under active development.

#### 6.3.1 Targeted nanoparticles for precision delivery

Given the significant therapeutic contradiction of FSP1/Nrf2 activation between sepsis and oncology, systemic administration of FSP1 agonists carries inherent safety risks. To address this, nanoparticle-based drug delivery systems represent a promising solution for tissue-specific and transient targeting. Recent pre-clinical advances have utilized liposomal or polymeric nanoparticles to encapsulate Nrf2 activators, CoQ_10_, or FSP1-stabilizing agents (127–131). However, these findings have not yet been validated in sepsis models; research in these areas holds promise as a future approach to targeted sepsis treatment. Developing transient, organ-targeted nanomedicines represents a key strategy to maximize anti-ferroptotic protection in septic tissues while avoiding the risk of systemic, long-term oncogenesis.

#### 6.3.2 Clinical ferroptosis biomarkers for patient stratification

To clinically translate FSP1-targeted therapies, reliable biomarkers are essential to monitor ferroptotic burden and stratify septic patients. Key clinical candidates include Plasma malondialdehyde (MDA), 4-hydroxynonenal (4-HNE), serum ferritin, hepcidin and soluble transferrin receptor (sTfR) (132–134). Monitoring these circulating markers will help identify patients experiencing active lipid peroxidation, thereby defining the optimal therapeutic window for FSP1-targeted interventions.

#### 6.3.3 Dual GPX4/FSP1 combination therapies

Because FSP1 and GPX4 operate as parallel, mutually compensatory cellular safeguards against lipid peroxidation, targeting a single pathway often triggers compensatory upregulation of the other, limiting monotherapy efficacy (15, 41). Consequently, combination therapies concurrently targeting both GPX4 and FSP1 pathways represent a highly synergistic strategy (116, 135). Therefore, in the treatment of infectious organ injury, combining physiological GPX4 restorers (such as selenium supplements or N-acetylcysteine to replenish glutathione) with FSP1 supportive interventions (such as CoQ_10_ pre-stimulation or transient Nrf2 activation) may provide multi-level protection, not only by raising the cells’ antioxidant threshold but also by allowing for lower doses of each individual agent, thereby reducing potential side effects.

## 7 Conclusion and future perspectives

Accumulating evidence indicates that FSP1-mediated ferroptosis suppression plays a critical role in the pathogenesis and progression of sepsis-associated organ injury. During sepsis, oxidative stress, cytokine storms, and metabolic derangements trigger intracellular iron dyshomeostasis and lipid peroxidation accumulation, thereby inducing ferroptotic cell death in vital organs—including the heart, liver, kidney, lung, and brain—and exacerbating organ dysfunction. As a pivotal ferroptosis repressor independent of the GPX4 system, alterations in FSP1 expression and activity are tightly correlated with sepsis severity and tissue damage, suggesting its potential as a latent biomarker for disease assessment and prognostic stratification. Current studies demonstrate that modulating FSP1 expression or activity alleviates organ injury and improves survival rates in experimental models, underscoring that targeting the FSP1-ferroptosis pathway represents a promising therapeutic strategy for sepsis. Nevertheless, several challenges remain: the precise regulatory mechanisms of FSP1 in sepsis are not yet fully elucidated, and current interventions are largely confined to animal models and lack organ-specific delivery systems, posing risks of off-target effects and safety concerns. In addition, the oncogenic risk associated with systemic Nrf2/FSP1 activation represents a critical safety barrier that remains unaddressed.

Future research should focus on: (1) further elucidating the sepsis-specific expression patterns of FSP1 regulators, particularly validating the transcriptional repression of FSP1 by IRF1, as well as the *in vivo* relevance of speculative mechanisms—such as CD36-mediated ubiquitination, ALKBH5-mediated m6A modification, and SENP3-mediated de-SUMOylation—in native sepsis models, thereby evaluating their clinical utility as early warning indicators; (2) developing FSP1 agonists with enhanced specificity and safety profiles, or utilizing transient-release, tissue-specific delivery platforms to maximize anti-ferroptotic protection in injured organs while avoiding the systemic, long-term activation that could otherwise promote tumor progression; (3) strengthening translational research through well-designed clinical trials to rigorously evaluate the safety, efficacy, and risk-benefit ratios of existing therapeutic modalities (such as Nrf2 agonists or CoQ_10_ supplementation).

Overall, the FSP1-mediated anti-ferroptotic pathway represents a critical mechanism in septic organ injury and a prospective target for clinical diagnosis, monitoring, and therapeutic innovation.
